# Roadside ditch macroplastic and other litter dataset in the Finger lakes region across land uses and COVID-19 pandemic

**DOI:** 10.1016/j.dib.2021.107425

**Published:** 2021-09-24

**Authors:** Olivia Pietz, Mary Augenstein, Christine B. Georgakakos, Kanishka Singh, Miles McDonald, M. Todd Walter

**Affiliations:** a Department of Natural Resources and the Environment, Cornell University, United States; b Department of Chemistry and Chemical Biology, Cornell University, United States; c Department of Civil and Environmental Engineering, Cornell University, United States; d Department of Biological and Environmental Engineering, Cornell University, United States; e Department of Earth and Atmospheric Sciences, Cornell University, United States

**Keywords:** Macroplastic, Plastic pollution, Terrestrial environment, Roadside ditches, Litter

## Abstract

Litter was collected from 12 roadside ditches in the Finger lakes Region of New York State over two sampling periods: pre-COVID-19 pandemic and during COVID-19 pandemic. Litter pieces were washed in DI water, oven dried, massed and plastic-type visually determined. Macroplastic data was analysed to assess the impact of land use, traffic, and COVID-19 variables on macroplastic accumulation on a piece, mass, and plastic-type basis. These data are all litter pieces collected, including both plastics categorized as 1 through 7 in the RIC resin classification codes as well as non-plastic litter. These data have wide-ranging reuse potential, as terrestrial microplastic accumulation is not well documented. These data could be compared with other litter accumulation across regions, specifically to assess total environmental macroplastic loading and enable contaminant mitigation strategies. These data also have direct application to modelling and transport of macroplastics into surface water bodies as a result of road ditch sampling locations. Macroplastic accumulation data across varying land uses, traffic, and COVID-19 conditions has been published [1].

**Specifications Table**

**Table:** 

Subject	Environmental Science: Pollution
Specific subject area	Roadside litter was collected from roadside ditches to assess accumulation of macroplastics.
Type of data	Table
How data were acquired	Data were collected June through December (2019) and August through November (2020). Plastic type was visually determined and categorized based on Resin Identification Code (RIC) established by the Plastic Industry Association [2]. Litter was washed in DI water and oven dried prior to recoding mass to remove sediment and debris associated with litter pieces. Sampling locations were repetitively revisited to enable calculation of macroplastic accumulation in ditches.
Data format	Raw
Parameters for data collection	Dataset was designed to enable macroplastic accumulation analysis across land uses and road traffic. Sampling sites were chosen to spread the 4 most common land uses in the Finger lakes region: agriculture, commercial, residential, and forested. Some non-plastic data are also included.
Description of data collection	Data were collected from 12 roadside ditches in the Finger lakes region of New York State. All litter was washed and oven dried prior to mass analysis. Three replicate ditches in each of 4 land uses were selected in the Tompkins Country region. Ditches were repetitively sampled to enable calculation of macroplastic accumulation rates.
Data source location	Institution: Cornell University City/Town/Region: Tompkins County, New York Country: United States Latitude and longitude for collected samples/data: Table 1 (Pietz et al*.,* 2021)
Data accessibility	These data are publicly hosted on Mendeley Data. Repository name: Mendeley Data Direct URL to data: https://doi.org/10.17632/sxfdf37wp4.1 https://data.mendeley.com/datasets/sxfdf37wp4/1
Related research article	Pietz, O., Augenstein, M., Georgakakos, C. B., Singh, K., McDonald, M., & Walter, M. T. (2021). Macroplastic accumulation in roadside ditches of New York State's Finger lakes region (USA) across land uses and the COVID-19 pandemic. *Journal of Environmental Management*, *298*, 113524. https://doi.org/10.1016/j.jenvman.2021.113524

**Value of the Data**

• Macroplastic accumulation in the terrestrial environment is not well documented, making mitigation of this contaminant difficult.
• This dataset is useful for policy makers by guiding plastic-focused decisions, local conservation organizers by reccomending mitigation locations, and other researchers interested in modelling macroplastic and litter accumulation across a wider region.
• These data are well suited for modelling risk of macroplastic transport through roadside ditches and into surface water systems. Pooled with other similarly collected data, these data begin to describe macroplastic loads in the terrestrial environment.

## 1 Data Description

*Dataset:* Each piece of litter collected during sampling comprises one row of the dataset. Information recorded for each piece include: mass, length, width, thickness, plastic type, and qualitative plastic use description. Sites were repetitively sampled, allowing calculation of litter accumulation since initial sampling site clearing. Round 0 indicates the date that all pre-existing plastics were purged from the sites, so that accumulation rates could be calculated.

## 2 Experimental Design, Materials and Methods

Collection of this dataset was inspired by a question around current plastic bag policies implemented in New York State. To wholistically assess the impact of macroplastic contamination in the Finger lakes region of New York state, these data document individual plastic piece masses, types, and sizes found in roadside ditches. Sampling ditches were chosen across 4 land uses (agriculture, commercial, residential, forested), with 3 replicates within each land use using the Tompkins country land use maps [3]. Traffic (cars/h) was recorded during sampling. Sampling occurred before the COVID-19 quarantine restrictions (2019) and during COVID-19 restrictions (2020). Analysis was completed on accumulation of mass and plastic pieces across the variables of land use and COVID-`9 period and proportions of plastic pieces within each plastic type collected.

Ditch characteristics (supplementary material of Pietz et al. (2021)) were collected at each site, with observations of changes in ditch characteristics (i.e. mowing, scraping) noted. These changes in ditch characteristics may have impacts on types and number of plastics collected after ditch changes occurred. Future studies may wish to more specifically design experiments around these ditch changes to monitor impact on plastic shredding and transport.

Individual litter pieces were washed in DI water and oven dried for a minimum of 24 h before mass analysis. Length, width, and thickness were measured using ruler and callipers. Plastic type was visually determined according to the RIC system [2].

A 60 m continuous stretch of roadside ditch was selected for each sampling reach. 1 m on either side of the center of the ditch was sampled, generating a 120 m^2^ sampling area. Ditches were not sampled during winter months due to inconsistent snow coverage and potential transport of macroplastics by snow plows.

## Ethics Statement

These data were collected without direct human or animal impact.

## CRediT Author Statement

**Olivia Pietz:** Project administration, Writing – original draft preparation; **Mary Augenstein:** Project administration, Data curation, Data visualization, Data analysis, Writing – original draft preparation; **Christine Georgakakos:** Conceptualization, Methodology, Visualization, Data analysis, Writing – original draft preparation, Writing – review & editing; **Kanishka Singh:** Conceptualization, Data analysis, Writing – original draft preparation, review, and editing; **Miles McDonald:** Project administration, Visualization, Writing –review & editing; **M. Todd Walter:** Writing – review & editing.

## Declaration of Competing Interest

The authors declare no conflicts of interest.

## References

[1] Pietz O., Augenstein M., Georgakakos C., Singh K., McDonald M., Walter M.T. (2021). Macroplastic accumulation in roadside ditches of New York State’s Finger Lakes region (USA) across land uses and the COVID-19 pandemic. J. Environ. Manag..

[2] Plastic Industry Association. (1988). Resin identification codes active standard ASTM D7611/D7611M. *Book of Standard Volumes 08.03.*

[3] Tompkins Co. (2020). Planning—Natural Resources Inventory www.tompkinscountyny.gov. https://tompkinscountyny.gov/planning/nri-nri2.

